# Validation of the Self-Assessment of Treatment Questionnaire among Patients with Postherpetic Neuralgia

**DOI:** 10.1155/2012/621619

**Published:** 2012-08-13

**Authors:** Kathleen W. Wyrwich, Ariane K. Kawata, Christine Thompson, Stefan Holmstrom, Malcolm Stoker, Ingela Wiklund

**Affiliations:** ^1^United BioSource Corporation, 7101 Wisconsin Avenue, Suite 600, Bethesda, MD 20814, USA; ^2^Astellas Pharma Global Development, 2350 AC Leiderdorp, The Netherlands; ^3^Astellas Pharma Global Development, Middlesex TW18 3AZ, UK; ^4^United BioSource Corporation, London W6 7HA, UK

## Abstract

*Introduction*. A five-item Self-Assessment of Treatment (SAT) was developed to assess improvement and satisfaction with treatment associated with the application of a novel high concentration 8% capsaicin topical patch in clinical trials in patients with postherpetic neuralgia (PHN). This study evaluated the item performance and psychometric properties of the SAT. *Methods*. The SAT, Brief Pain Inventory, SF-36v2, Short-Form McGill Pain Questionnaire, and Patient and Clinician Global Impression of Change (PGIC; CGIC) scores were measured in two 12-week Phase 3 clinical trials. Factor analysis assessed the underlying factor structure, followed by examination of the reliability and validity of the multi-item domain. *Results*. Pooled data from 698 patients completing SAT after 12 weeks of treatment were analyzed. A one-factor model combining three of the five items emerged as the optimal solution. Internal consistency reliability of this treatment efficacy factor was high (Cronbach's alpha = 0.89). Construct validity was demonstrated by moderate to high correlations with change in other study endpoints. SAT mean scores consistently discriminated between patient change groups defined by PGIC and CGIC. *Conclusions*. The measurement properties of the three-item version of SAT are valid and reliable for assessment of treatment with a high concentration capsaicin patch among patients with PHN.

## 1. Introduction

Postherpetic neuralgia (PHN) is a rare and debilitating complication of an acute herpes zoster (shingles) episode and is defined as pain that persists more than three months after the zoster skin lesions (rash) have healed [1]. Typically, individuals with PHN develop severe pain in the area of the body, usually the trunk, where shingles occurred. This debilitating pain, described as burning, sharp, jabbing, deep, and aching, can persist for months or years and is often not responsive to oral analgesics [2]. Recently, the European Medicines Agency (EMA) and the US Food and Drug Administration (FDA) approved the use of a high concentration capsaicin topical patch 8% (QUTENZA) for the treatment of peripheral neuropathic pain in nondiabetic adults, either alone or in combination with other medicinal products for pain (EU label), and the management of neuropathic pain associated with postherpetic neuralgia (US label), based on the results from two Phase 3 randomized, double-blind, dose-controlled trials in subjects with PHN [3, 4]. Most commonly known as the pungent component of hot chili pepper, capsaicin in high concentrations like the 8% topical patch is a transient receptor potential vanilloid 1 (TRPV1) agonist that is useful in relieving pain. In the body, the TRPV1 receptors are expressed in sensory neurons that detect noxious painful stimuli. Therefore, the agonist effect of capsaicin at VR1 receptors results in the defunctionalization of sensory nerve endings [4]. 

For the past decade, the Initiative on Methods, Measurement, and Pain Assessment in Clinical Trials (IMMPACT) have developed consensus reviews and recommendations for improving the design, execution, and interpretation of clinical trials of treatments for pain. At their first meeting in November 2002, agreement was reached on the core outcome domains that should be considered by investigators conducting clinical trials of the efficacy and effectiveness of treatment for chronic pain. The six recommended core domains were: (1) pain, (2) physical functioning, (3) emotional functioning, (4) participant ratings of improvement and satisfaction with treatment, (5) symptoms and adverse events, and (6) participant disposition [5].

Pain relief and patient satisfaction are distinct concepts identified by IMMPACT as central to evaluating treatment of chronic pain. Pain relief measures are used to determine whether the patient has actually benefited from an intervention and provide valuable information on how effectively pain is being managed. In contrast, patient satisfaction measures capture the personal evaluation of the intervention provided [6]. The American Pain Society (APS) Satisfaction Survey was tested to evaluate the relationships among the survey items, and whether the items are related to satisfaction [7]. The APS survey demonstrated a weak relationship between pain intensity and satisfaction, and satisfaction was influenced largely by effectiveness of medication, independent of pain intensity [7]. These results highlight that a satisfaction survey related to the effectiveness of a pain medication is an important measurement tool.

In planning the clinical trials for this novel pain treatment, satisfaction surveys in pain were reviewed to evaluate whether an existing instrument could be used in clinical trials to measure the IMMPACT core domain of patient satisfaction. The APS Satisfaction Survey focuses on pain management in general practice, but is not directly related to satisfaction with medication. Another existing survey examined was the Pain Treatment Satisfaction Scale (PTSS) [8], which measures satisfaction in patients receiving treatment for either acute or chronic pain. However, it was not designed for use in clinical trials, as it evaluates satisfaction with medical care received as well as pain medication. A third instrument that was considered for measuring patient satisfaction was the Patient's Global Impression of Change (PGIC) [9]. However, PGIC asks the patient to rate change in their overall status, which relates to multiple domains of health, rather than only assessing satisfaction with the pain treatment. Therefore, this instrument is too generic to fully describe which domains are impacted most by this novel treatment for pain. Although there were a variety of instruments capturing patient satisfaction, no existing measures were identified as appropriate for assessing patient satisfaction with medication used to treat chronic pain in the context of a clinical trial.

This lack of a suitable pain satisfaction instrument for use in trials that measure multiple domains of importance to patients supported the development of a new instrument. A recent IMMPACT survey [10] also identified 19 relevant domains of patient-reported outcomes from the perspective of people who experience pain. In addition to pain relief, aspects of daily life related to functioning and wellbeing were identified as key areas affected by symptoms that should be targeted by treatment. The IMMPACT results stress the importance of including these domains when measuring treatment efficacy and pain relief, and they should also be taken into consideration in measuring satisfaction.

Therefore, to measure the IMMPACT-recommended domains of participant-reported improvement and satisfaction with treatment and incorporate aspects of daily life, the five-item Self-Assessment of Treatment (SAT) questionnaire was developed for use in the clinical trials evaluating a high concentration 8% capsaicin topical patch (Table 1). The SAT was developed based on this need for a clinically meaningful instrument meeting the properties of QUTENZA and the specific symptoms of PHN patients to be used in the clinical development program for QUTENZA, given that no existing treatment satisfaction instrument was identified that was deemed suitable for this work. This study examined the item performance and psychometric properties of the SAT to validate this instrument and enhance the future use of the SAT questionnaire. Standard quantitative methods were conducted using data from both of the Phase 3 registration trials to explore the factor structure, reliability, and validity of the SAT multi-item scales.

## 2. Methods

### 2.1. Study Objective

The primary objective of this study was to evaluate the psychometric properties of the SAT questionnaire as part of the validation of this instrument in patients with moderate to severe neuropathic pain secondary to PHN.

### 2.2. Study Design

This analysis used data collected as part of Studies C116 and C117, two Phase 3 randomized, double-blind, controlled, multicenter clinical trials conducted by NeurogesX to evaluate the efficacy, safety, and tolerability of a high-concentration 8% capsaicin topical patch (640 mcg/cm^2^), for the treatment of PHN (clinical trial identifiers: NCT00115310, NCT00300222).

Subjects eligible for inclusion in the two studies were adults in good health with a diagnosis of PHN and at least six months since shingles vesicle crusting, with an average Numeric Pain Rating Scale (NPRS) score for PHN-associated pain of 3 to 9, inclusive, on a scale of 0 = no pain and 10 = worst possible pain, during the screening period (usually 14 days before Study Patch Application Visit). Exclusion criteria included subjects with other pain conditions (e.g., compression-related neuropathies, fibromyalgia, arthritis) or cognitive impairment that might interfere with judging PHN-related pain or completing pain assessments.

Patients received either the study medication or low-concentration capsaicin (3.2 mcg/cm^2^) patches for 12 weeks. Low-concentration capsaicin control patches were used in place of placebo patches to allow for effective blinding of the study, since topical capsaicin can produce a local erythema and a burning sensation. Study C116 included 52 centers in the US, and Study C117 was comprised of 61 study sites in the US and Canada.

The primary objective of the two PHN studies (C116 and C117) were assessment of capsaicin patch efficacy over 12 weeks. The primary efficacy variable in each clinical trials was the percent change in “average pain for the past 24 hours” NPRS scores from Baseline to Weeks 2–8. The NPRS item is one of many other pain items in the Brief Pain Inventory (BPI). Percent change and proportion of subjects with 30% and 50% decreases in NPRS scores from Baseline to Weeks 2–8 and Weeks 2–12 were key secondary efficacy measures. Other efficacy measures included change in other BPI items, Short-Form McGill Pain Questionnaire (SF-MPQ) pain intensity rating, Medical Outcomes Study Short Form-36 Heath Survey, version 2 (SF-36v2), Patient Global Impression of Change (PGIC), and Clinical Global Impression of Change (CGIC) scores from Baseline to Weeks 4, 8, and/or 12 (when measured at followup) and SAT at Week 12 (end of study).

### 2.3. Study Measures

#### 2.3.1. Patient-Reported Outcome (PRO) Measures

**Brief Pain Inventory (BPI)**

The BPI [11] provides an index of pain severity, pain relief, and the effects of pain on the subject's ability to function. The standard BPI questionnaire includes nine items, but a modified version of the BPI (Short Form [12, 13]) was used in Studies C116 and C117. The BPI was completed at Screening, Week 8, and Termination Visit (Week 12).

The BPI administered included four questions on pain levels, where subjects were asked to rate their pain on a scale of 0 (no pain) to 10 (worst possible pain) in response to (1) pain at its *worst* in the last 24 hours; (2) pain at its *least* in the last 24 hours; (3) pain *on average* in the last 24 hours (NPRS item); (4) pain *right now*. An additional question asked subjects to rate the level on interference of their pain with general activity, mood, and other activities of daily living on a scale of 0 (does not interfere) to 10 (completely interferes). Pain interference was assessed in seven areas: (1) general activity; (2) mood; (3) walking ability; (4) normal work (includes both work outside the home and housework); (5) relations with other people; (6) sleep; (7) enjoyment of life.

Data on the “pain now” item was collected at all study visits (Screening, Baseline (Week 0), Week 4, Week 8, and Termination Visit (Week 12)). Subjects also recorded NPRS scores for “average pain for the past 24 hours” daily in a take-home diary beginning on the evening of the Study Patch Application Visit (Day 0) through the evening before the Week 12 visit.

**Short-Form McGill Pain Questionnaire (SF-MPQ)**

The SF-MPQ [14] asks subjects to identify their Present Pain Intensity (PPI) on a scale of 0 (no pain) to 5 (excruciating). The SF-MPQ also includes sensory and affective pain descriptors. The SF-MPQ was administered at Screening, Week 8, and Termination Visit (Week 12).

**Medical Outcomes Study Short Form-36 Heath Survey, Version 2 (SF-36v2)**

The SF-36v2 is an assessment of overall health and wellbeing rated in eight areas, including overall health, ability to perform various physical activities, emotional problems, social functioning, vitality, and pain in the previous 4 weeks [15, 16]. Scores range from 0–100, with higher scores indicating better health status. The SF-36v2 was administered at Screening and Week 8.

#### 2.3.2. Subjective-Rated Measures of Treatment Effectiveness

**Patient Global Impression of Change (PGIC) and Global Impression of Change (CGIC)**

The PGIC and CGIC addressed change in the severity of a patient's illness over a particular time interval. In the C116 and C117 clinical trials, the reference time period was “after receiving study treatment.”

The PGIC was patient-reported, and asked the subject to “indicate how you feel now, compared to how you felt before receiving treatment in this study” on a 7-point scale of −3 (very much worse), 0 (no change), to +3 (very much improved). This rating scale permitted a global evaluation of the *patient's *impression of change in their condition since admission to the study. The PGIC was completed at all three visits (Weeks 4, 8, and 12) in Studies C116 and C117, following the Study Patch Application Visit.

The CGIC was completed by the study investigator, who was asked to compare “how the subject appears to you now, compared to how they appeared to you before receiving treatment in this study” on a 7-point scale of −3 (subject very much worse), 0 (no change), to +3 (subject very much improved). This rating scale permitted a global evaluation of the *clinician's* impression of change in the patient's condition since admission to the study. In Study C117, the CGIC was collected at all study visits after Baseline (Weeks 4, 8, and 12); the CGIC was not completed in Study C116.

**SAT Questionnaire**

Subjects were asked to assess capsaicin patch treatment using the SAT questionnaire at the Termination Visit (Week 12). The SAT evaluation form included five questions with three- or five-point response options (Table 1). The items included assessments after treatment in the study for three areas (pain relief; activity level; quality of life) and two additional items regarding (1) whether the patient would undergo the treatment again, and (2) a comparison of the study treatment to previous treatments for pain.

For each question, the subject checked a box on a five-point scale, where the middle option (0) indicated a neutral response and the lower (−2) and higher (+2) options indicated a negative or positive response, respectively. For example, SAT Item 1 asked the patient “How do you assess your pain relief after treatment in this study?” with the response options of “I feel my pain is much worse” (−2), “somewhat worse” (−1), “no better and no worse” (0), “somewhat better” (1), and “much better” (2). In Study C116, Question 4 “Would you undergo this treatment again?” was administered to subjects with only three response options: “No, absolutely not” (−2), “Unsure” (0), and “Yes, definitely” (2).

### 2.4. Statistical Analyses

Post hoc analyses of the SAT and other PROs were performed on the intent-to-treat (ITT) population, which included all subjects enrolled in the C116 or C117 studies who were randomized, received the study drug, and had at least three days of nonmissing “average pain for the past 24 hours” NPRS scores for the calculation of the Baseline NPRS score. The analyses incorporated the patient population for whom Termination Visit (Week 12) data were available, as this was the only time in both studies that SAT data were collected. The schedule of visits and study measures used in this analysis are summarized in Table 2.

The psychometric analyses focused on the factor structure, reliability, and validity of the SAT in the C116 and C117 datasets. Psychometric properties of the SAT were first assessed using data from Study C116; replicability of the results for SAT psychometric properties was investigated using data from Study C117. Results from analyses using the pooled samples are presented herein, given the representativeness of this larger dataset and the replicated psychometric properties demonstrated by each individual study. SAS statistical software version 9.2 (SAS Institute Inc., Cary, NC, USA) and MPlus version 5.21 (Muthén & Muthén, Los Angeles, CA, USA) were used to conduct the analyses. All statistical tests were two-sided and used a significance level of 0.05 unless otherwise noted.

#### 2.4.1. Missing Values and Scoring Algorithms

For the SF-MPQ and SF-36v2, missing data were handled per the instrument developer's scoring instructions. NPRS scores for average pain in the last 24 hours were based on a daily take-home diary; baseline and Week 12 scores were computed as the average NPRS pain rating for 7 days prior to the visit. Observed data were used for analyses, with no additional imputations for missing data unless otherwise specified. As part of the exploratory nature of the analyses of the SAT's measurement properties, individual SAT items were analyzed separately, and composite subscale scores were generated. Two subscales informed by the confirmatory factor analysis (CFA) results were evaluated, reflecting items relating to treatment success (SAT Items 1, 2, and 3) and treatment satisfaction (SAT Items 4 and 5) computed as the arithmetic average of respective items' responses scores (Table 1).

#### 2.4.2. Descriptive Statistics

**Sociodemographic and PRO Measures**

Descriptive statistics (mean, median, standard deviation (SD), minimum, and maximum for continuous variables and frequencies for categorical variables) for patients in the pooled sample were reported. Age, gender, race/ethnicity, height, and weight were evaluated at screening. Descriptive statistics (mean, SD, median, minimum, and maximum) for PRO subscale and component scores were examined for the overall sample. PRO measures included NPRS “pain now” and “average pain in the last 24 hours” ratings at screening and baseline (Week 0), average scores for the subscales of the SF-36v2 and SF-MPQ pain intensity at screening, BPI pain scores and composite pain interference scores at screening, SAT items and subscales, PGIC for Studies C116 and C117, and CGIC for Study C117 at Week 12.

**SAT Factor (Scale) Structure**

After examining the Spearman inter-item correlations to assess the extent to which the five SAT items correlated with each other, an exploratory factor analysis (EFA) and a CFA using a structural equation modeling (SEM) approach was conducted to evaluate the factor (scale) structure of the SAT and fit of the items within the hypothesized scale. The EFA and CFA were performed using the five items comprising the SAT at Week 12. In the SEM approach, parameter estimates were generated based on analysis of the actual covariance matrices representing the relationships among SAT items and the estimated covariance matrices of the measurement model. Measurement models for one and two domains were developed, with each item loading on its respective scale. In addition, factor solutions with eigenvalues near or greater than 1.0 were examined, as well as the amount of variance accounted for by the resulting factor structure. The overall fit of each model was assessed, as well as the magnitude of the item factor loadings.

In the CFA analyses, several fit statistics were used to provide information about the adequacy of the model to explain the data. In general, the model was considered to explain the data well if the comparative fit index (CFI) was ≥0.90. The standardized root mean residual (SRMR) measures the mean absolute difference between the observed and model-implied correlations; values <0.1 were considered acceptable [17]. Finally, the root mean square error of approximation (RMSEA) is a measure of fit assessing the discrepancy between the predicted and observed data per degree of freedom; values <0.08 were considered acceptable [18] and the 90% CI for the RMSEA should be narrow, thereby giving additional confidence in the estimate. Adequacy of item fit was also assessed through the examination of modification indices, item residual correlations, and item factor loadings.

**Internal Consistency Reliability**

Internal consistency reliability is a measure of the consistency of results across individual items on the same instrument. Internal consistency reliability of the SAT was evaluated using Cronbach's alpha [19] to calculate coefficients for the total instrument using data for the Termination Visit (Week 12), with a value greater than 0.70 denoting a more homogeneous instrument, offering acceptable reliability [20].

**Construct Validity**

Construct validity refers to the extent to which the instrument measures what it is intended to measure [20]. Construct validity of the SAT items and subscales were evaluated through the examination of the relationships between the SAT, subscales, and component scores of conceptually-related outcome measures using Spearman correlation coefficients. It was expected that patients reporting higher improvements on the PGIC and CGIC at Week 12 would also score better in the SAT items and subscale scores. In addition, correlations between the SAT item and subscale scores and pain at the moment of responding were also explored, using the different pain questions available at Week 12: NPRS pain now and the last 24-hour average; BPI pain at worst and at least, and pain interference assessments; pain dimensions and present pain intensity of the SF-MPQ; three SF-36v2 scores for physical functioning, pain, and vitality domains most closely associated with pain. The latter were not recorded at Week 12, so change from Baseline to Week 8 was used instead. The resulting SF-36v2 subscale mean scores were also compared to the means for the US general population.

**Known-Groups/Discriminant Validity**

The ability of the SAT items and composite and subscale scores to discriminate between groups of patients according to levels and changes of symptom severity was also evaluated. Discriminant or known-groups validity was assessed using analysis of variance (ANOVA). These analyses provided a test of whether there were significant differences in mean SAT scores for different amounts of change based on other PRO measures. The ANOVAs were performed comparing mean SAT items and composite and subscale scores for the relevant time points and by groups defined by the following variables: (1) patient- and clinician-reported change groups created by PGIC and CGIC using the seven levels of change, and (2) high (NPRS ≥ 7) and lower (NPRS < 7) pain patients at baseline [21].

**Concurrent Validity**

To evaluate concurrent validity of the SAT, all items and the composite and subscale scores were used. According to the SAT responses, three response levels were created: (1) patients who improved (much better and somewhat better, or probably and definitely would undergo treatment again); (2) patients with no change (no better and no worse, or unsure about undergoing treatment again); (3) patients who worsened (much worse and somewhat worse, or probably and definitely not undergo treatment again). NPRS and other BPI item change scores were compared by SAT response groups using ANOVA models; average change scores from baseline to Week 12 in the pain reported by the NPRS pain now and average 24-hour pain and from screening to Week 12 for BPI worst and least pain items were evaluated.

## 3. Results

### 3.1. Patient Characteristics

A total of 698 patients from the ITT populations of Studies C116 (*N* = 349) and C117 (*N* = 349) were included in the current SAT analyses. Patient characteristics (age, sex, and race/ethnicity) for the patient population pooled across the two trials were similar between treatment groups (Table 3). Patients were predominantly white, with slightly more female patients (54.3%), and a mean age of 71 years (range 21–94 years).

PRO scores prior to the start of treatment provide an overall description of patient condition (Table 4). At screening, NPRS “pain now,” BPI pain ratings and pain interference, SF-MPQ pain rating, and SF-36 bodily pain subscale scores consistently indicated that patients reported noticeable levels of pain prior to study treatment. The mean NPRS “pain now” rating at screening was 4.7 (SD = 2.2) with a median rating of 5 on the 0–10 scale. BPI scores for pain ratings and pain interference on a scale of 0–10 also indicated the presence of pain and interference from pain in most patients. SF-MPQ had a mean present pain intensity rating of 2.1 (SD = 0.9) and a median of 2 on a 0–5 scale at Screening. Average SF-36v2 subscale scores for bodily pain (mean = 44.0, SD = 18.9) were lower than other SF-36 subscales mean scores. Moreover, all mean subscale scores were below the respective US general population averages, indicating worse than average health [16].

### 3.2. Psychometric Properties of the SAT

#### 3.2.1. Descriptive Statistics

Descriptive statistics for SAT items and composite and subscale scores at Week 12 are reported in Table 5 for the blinded data. Positive SAT scores corresponded to patient assessment of improvement at the completion of the study. Mean scores for SAT items ranged from 0.4 (activity level) to 1.0 (undergo treatment again), relating to an average rating between neutral and somewhat improved. On Items 1 to 3, patients reported pain relief (22.1%) and quality of life (16.3%) as “much better” and feeling “much more active” (12.3%). Over half of the patients responded that they would definitely undergo the treatment again (SAT Item 4; 51.0%). It is important to note that in Study C116, SAT Item 4 was administered to subjects with only three response options rather than a five-level response scale, which may inflate these results. Nearly one quarter responded that they preferred the study treatment to previous treatments they had received (SAT Item 5; 25.6%). Very few patients responded at the lowest possible score on SAT items; the most frequent were 5.9% on SAT Item 4 (undergo treatment again) and 5.6% on Item 5 (compared to previous treatment).

#### 3.2.2. Inter-Item Correlations

Spearman inter-item correlations assessed the extent to which the five items of the SAT correlated with each other and with the composite scores (data not shown). Items 1 (pain relief), 2 (activity level), and 3 (quality of life) were strongly correlated with each other, and moderately correlated with Items 4 (undergo treatment again) and 5 (compared to previous treatment). Correlations among the first three items ranged from 0.67 to 0.77 (all *P* < 0.0001), while their bivariate relationships with Items 4 and 5 were weaker, ranging from 0.35 to 0.60 (*P* < 0.0001). There was a moderate correlation between Items 4 and 5 (*r* = 0.51, *P* < 0.0001).

#### 3.2.3. SAT Factor (Scale) Structure

One- and two-factor measurement models of the SAT were developed using the pooled dataset (Study C116 and C117 combined) to evaluate item loadings and overall model fit (Table 6). Factor solutions that had eigenvalues near or greater than 1.0 and accounted for substantial amounts of the variance were considered.

**Exploratory Factor Analysis**

Exploratory one- and two-factor models were evaluated to determine the factor structure of the SAT (Table 6). The one-factor solution, including all five SAT items, had an eigenvalue of 3.26, and the model explained 65% of the variance in the SAT. Factor loadings ranged from 0.47 to 0.85, suggesting that all five items were related to the overall treatment construct. EFA results showed that factor loadings were largest for SAT Items 1 to 3, with all loadings >0.80; loadings for the other SAT items were acceptable, but slightly lower with a loading of 0.47 for SAT Item 4 and 0.65 for SAT Item 5.

A two-factor exploratory model was also specified to evaluate the tenability of extracting a second factor (Table 6). Eigenvalues in the two-factor model were 3.26 for the first factor and 0.79 for the second factor. The proportion of variance explained by the first factor was 65%, and total variance explained by the model was 81%; the addition of a second factor in the model accounted for an additional 16% of variance in SAT items. Factor loadings showed a clear demarcation between factors, with SAT Items 1, 2, and 3 loading on the first factor (treatment effects), and SAT Items 4 and 5 loading of a second factor (treatment satisfaction).

**Confirmatory Factor Analysis**

Based on the EFA results, confirmatory models were performed to formally test the one- and two-factor structures (Table 6). In the single factor model, the five SAT items were specified to load onto the first factor. General results for the CFA model were the same as reported previously for the one-factor EFA model. The chi-square test for model fit was highly significant (*χ* ^2^(df = 5) = 92.83, *P* < 0.0001). Model fit statistics showed good fit (CFI = 0.95) and relatively low residuals (SRMR = 0.048); RMSEA suggested a slightly worse fit (RMSEA = 0.16, 90% CI = 0.13–0.19).

In the second model, SAT Items 1, 2, and 3 were specified as loading on the first factor, and SAT Items 4 and 5 as loading on the second factor. The chi-square test of model fit for the two-factor CFA was significant (*χ* ^2^(df = 4) =29.19, *P* < 0.0001). Model fit was very good (CFI = 0.99) with small residuals (SRMR = 0.024; RMSEA = 0.10, 90% CI = 0.07–0.13), indicating that the model adequately explained the data; RMSEA suggested a slightly worse fit than other fit indices. Loadings for the prespecified factor structure were generally large and consistent with the EFA results. Factor loadings for SAT Items 1, 2, and 3 with Factor 1 were 0.82, 0.85, and 0.93, respectively, and 0.59 for SAT Item 4 and 0.84 for SAT Item 5 on the second factor. Although the two factors were strongly correlated (*r* = 0.75), this two-factor solution created the best structure for interpretable SAT composite scale scores.

#### 3.2.4. Internal Consistency Reliability

Cronbach's alpha was used to examine internal consistency reliability for the two SAT subscales using combined patient populations from the two trials at the Termination Visit (Week 12). The SAT subscale comprised only of SAT Items 1 to 3 (pain relief, activity level, and quality of life) evaluating treatment effectiveness had excellent reliability, with an alpha of 0.89. A separate subscale made up of SAT Items 4 and 5 (undergo treatment again, compared to previous treatment) evaluating treatment satisfaction had an alpha of 0.66.

#### 3.2.5. Validity

**Construct Validity**

Construct validity of the SAT was assessed by examining relationships between SAT items and subscale scores with conceptually-related outcome measures using Spearman correlation coefficients (Table 7). Outcome measures included PGIC and CGIC at Week 12, change scores between Baseline (Week 0) and Week 12 for pain now and average 24-hour pain, change scores between Screening and Week 12 for BPI pain and interference and SF-MPQ pain dimensions, and change scores between screening and Week 8 for SF-36v2 physical functioning, bodily pain, and vitality subscales.

Moderate to large positive correlations were observed between SAT items and scores and PGIC and CGIC (Study C117 only) at Week 12 (Table 7). These positive relationships indicated that improvements based on global impressions of change were related to better evaluation of the study treatment at Week 12. All correlations reached statistical significance, and a similar pattern was found with both the patient and clinician assessments. Correlations between SAT items and PGIC in the combined sample ranged from 0.44 to 0.90 (all *P* < 0.0001). In Study C117, correlations with CGIC ranged from 0.48 to 0.85 (all *P* < 0.0001). SAT Item 1 (pain relief) was the most strongly related to PGIC (*r* = 0.90, *P* < 0.0001) and CGIC (*r* = 0.85, *P* < 0.0001). Correlations between SAT Item 4 (undergo treatment again) with PGIC (*r* = 0.44, *P* < 0.0001) and CGIC (*r* = 0.48, *P* < 0.0001) were quite smaller, although moderate in magnitude and statistically significant.

Among SAT composite scale scores, the three-item subscale comprised of pain relief, activity level, and quality of life had the largest correlations with patient (*r* = 0.89, *P* < 0.0001) and clinician (*r* = 0.83, *P* < 0.0001) assessments. The two-item subscale made up of SAT Items 4 and 5 was more weakly related to PGIC (*r* = 0.61, *P* < 0.0001) and CGIC (*r* = 0.64, *P* < 0.0001).

Overall, SAT was moderately correlated with changes in pain-related outcomes (Table 7). Statistically significant negative correlations indicated that better evaluation of study treatment was generally associated with reduction in pain over the study period. Correlations between SAT items and NPRS pain now and average 24-hour pain change scores ranged from −0.30 to −0.69 (all *P* < 0.0001). Changes in BPI pain at worst (*r* = −0.28 to −0.64, all *P* < 0.0001) and at least (*r* = −0.27 to −0.52, all *P* < 0.0001) in the last 24 hours and SF-MPQ present pain intensity scores (*r* = −0.20 to −0.45, all *P* < 0.0001) showed a similar range of correlations with SAT items. As expected, SAT pain relief (Item 1) consistently showed the strongest relationships with pain change scores. Associations between SAT activity level (Item 2) and quality of life (Item 3) with changes in pain were slightly smaller. The weakest relationships were found with SAT items related to treatment satisfaction (Items 4 and 5). Weaker associations were observed between SAT items and changes in BPI pain interference with general activity, mood, and other activities of daily living and SF-MPQ pain intensity ratings for sensory and affective descriptors (data not shown). The majority of these correlations were small, with none larger in magnitude than −0.40.

Among the SAT composite scale scores, the treatment effect subscale (pain relief, activity level, and quality of life) showed stronger relationships with changes in pain based on NPRS, BPI, and SF-MPQ items than the two-item treatment satisfaction subscale.

SAT items were more related to SF-36v2 bodily pain (*r* = 0.27 to 0.43, all *P* < 0.0001) than physical functioning (*r* = 0.14 to 0.25, all *P* < 0.001 or lower) or vitality (*r* = 0.09 to 0.25, all *P* < 0.05 or lower) subscales. Also, correlations between SAT treatment effect items (pain relief, activity level, and quality of life) and changes in health status were consistently larger than with SAT items related to satisfaction (undergo treatment again, compared to previous treatment). A similar pattern was obtained for SAT subscale scores with changes in SF-36v2 health status domains.

**Discriminant/Known-Groups Validity**

Known-groups analyses examined the ability of SAT items and subscale scores at Week 12 to discriminate between patient groups using patient- and clinician-reported change at Week 12 and NPRS pain levels at Baseline. Change groups represented global assessments of change over the study period using the seven response levels for PGIC and CGIC. NPRS pain ratings at Baseline were categorized as high (NPRS ≥ 7) and low (NPRS < 7) pain groups.

Significant differences in mean SAT items and scores between change groups based on PGIC and CGIC were identified. Results demonstrate an overall pattern of average SAT scores that differ as a function of response levels for global assessments of change (Table 8). SAT showed evidence of ability to discriminate between change levels based on patient and clinician global assessments; SAT scores had a pattern of least-squares means very close to zero, corresponding to the “no change” group for PGIC or CGIC, and increasingly positive mean SAT scores for global assessment improvement levels and corresponding negative mean SAT scores for worsening levels. The lowest response levels on the negative end of the PGIC/CGIC response scale had very small sample sizes; the “very much worse” and “much worse” responses were pooled for these analyses. The “slightly worse” global assessment level was still shown to have lower mean SAT scores than the “no change” group, and higher mean SAT scores than the “much worse/very much worse” group, as expected. Mean SAT scores significantly differed between change groups for SAT items and subscale scores (all *P* < 0.0001).

Some SAT scores showed evidence of ability to discriminate between patient pain level groups based on NPRS pain at Baseline (Table 9). Mean SAT scores for patients with high pain at Baseline were slightly lower than scores for lower pain patients. Based on *t*-test comparisons, patients with less pain at Baseline had significantly higher scores than those with high pain on SAT Items 1 (pain level; *P* < 0.01), 2 (activity level; *P* < 0.05), and 3 (quality of life; *P* < 0.05).

**Concurrent Validity**

Concurrent validity of SAT items and scores was evaluated using ANOVA models comparing average change scores from Baseline to Week 12 in pain reported by the NPRS pain now and average 24-hour pain, and Screening to Week 12 for BPI worst and least pain items by SAT response levels (patients who improved, no change, or worsened).

The degree of change in NPRS scores between Baseline and Week 12 of the study concurred with the classification into change groups based on SAT scores (Table 10). Negative change scores on NPRS pain now and average pain for the last 24 hours indicated decreases in pain over time, and SAT groups for “better,” “no change,” and “worse” were associated with varying amounts of change. Most NPRS change scores at all levels of SAT responses were negative, suggesting that patients overall had experienced a decrease in pain over the study period. On average, patients in the SAT improvement group had the largest negative change scores (two- to three-point decreases in pain); two-point changes in NPRS can be interpreted as reflecting important change [22]. Patients with no change had smaller change scores, generally less than a one-point mean decrease. Worsening reported on the SAT corresponded to either small negative change scores (small decreases in pain) or positive change scores, indicating more pain. Significant differences in NPRS measures were observed across the three SAT response levels for all items and scores (all *P* < 0.0001).

Change in pain based on BPI pain at its worst and pain at its least also reflected differences based on SAT response levels (Table 11). Magnitude of the change scores tended to be larger for BPI pain at its worst than for pain at its least in the last 24 hours, particularly for “better” and “no change” SAT groups. Similar to the NPRS outcomes, negative change scores corresponded to decreases in pain over time, and varying amounts of mean change on these BPI items were observed for SAT change groups. Average BPI change scores for the SAT “better” group were the largest, with decreases of up to four points. Both positive and negative mean changes were observed for the “no change” and “worse” groups. Based on the ANOVA models, differences in BPI change scores for pain by SAT groups were statistically significant (all *P* < 0.0001).

## 4. Discussion

Because the value and importance of therapeutic changes differ greatly among participants, as well as between patients and their clinicians [23], it is essential that chronic pain clinical trials directly measure patient-reported improvement and satisfaction with treatment [5].

The SAT was designed for use in clinical trials to assess the IMMPACT-recommended domain of improvement of patients with PHN and their satisfaction with treatment using a high concentration 8% capsaicin patch. Psychometric properties of the SAT examined in this study demonstrated that the first three items assessing improvement of pain relief, activity level, and quality of life had a strong factor structure, high internal consistency reliability, and moderate to strong construct validity with change in other study endpoints. Moreover, the three-item SAT scores consistently discriminated between patient change groups defined by the PGIC response categories in both studies and the CGIC responses used in Study C117. Two additional SAT items querying whether patients would undergo the treatment again and how the study treatment compares to previous medication or therapies for the patient's pain did not demonstrate a strong structural relationship with the other three items, although both are key components to understanding satisfaction with this treatment. These two items assess important treatment-related concepts for patients who must determine whether the positive attributes of a treatment outweigh any potential side effects; this determination is key in understanding whether current and future patients will adhere to and continue with treatment [5].

To provide useful and valid information, a satisfaction subscale needs to be reliable and valid, and should capture a meaningful concept. In this case, the measurement properties were weaker for the two-item satisfaction subscale in terms of internal consistency reliability and known-groups validity. The satisfaction subscale had lower construct and concurrent validity, and was also less responsiveness in detecting change. Moreover, although the factor analysis supported a two-factor solution, the magnitude of the second factor's loading did not support a clear and distinct concept.

Despite the favorable psychometric results displayed by the five SAT items—and specifically the three-item treatment effects scale—there are several limitations to these SAT items. First, the patient's response to the SAT items requires a 12-week retrospective assessment by the patient, requiring each to somehow remember their condition (pain, activity level, quality of life, etc.) before initiating treatment, and to mentally subtract this assessment from their current state to select the most appropriate response (Table 1). These retrospective assessments are known to be prone to recall bias [24], with a strong correlation to the current state and a weak relationship with the initial state. Second, the concepts of pain, activities, and quality of life can have broad meaning across a population of patients, and without more detailed terms (e.g., daily, strenuous, social, etc.) for each of these three concepts, it remains unknown what patients considered when making their assessments of change. Third, the response scales used in Item 4 differed between the two studies and could have contributed to this item's weaker psychometric properties compared to the other SAT items in these analyses. In addition, Item 5 asks patients to compare their treatment to previous medications or therapies for pain, but these comparator treatments remain unknown and may have greatly differed across the patients in these two clinical trials.

Although there is no publication describing the instrument development process for the SAT, these items assess key areas of treatment change (i.e., pain, activities, quality of life) recommended by IMMPACT [5] and are appropriate for use in clinical trial study and research settings to measure participant ratings of improvement and satisfaction with treatment stressed in the IMMPACT recommendations. However, the psychometric performance suggests that the questionnaire items could be further improved with additional patient input for item clarity, response option revision, and the associated recall period to better reflect pain-related treatment benefits. Future research grounded in patient input should examine the use of frequent cross-sectional assessments with a seven-day recall period to assess change in these concepts measured at Baseline and over time at study visits. Moreover, because activity level and quality of life are very broad terms, specific types of activity (e.g., daily, strenuous, social, etc.) may provide more interpretable measures. Similarly, asking about “quality of life” is also quite broad and nonspecific, and improved measurement of specific domains (e.g., emotional wellbeing, physical functioning, and social functioning) may also increase the usefulness of these patient ratings collected over time.

## 5. Summary

The ability of the SAT questionnaire to measure improvements and satisfaction with treatment in PHN clinical trials was psychometrically evaluated, with recommendations for future use.

## Figures and Tables

**Table 1:** Self-Assessment of Treatment (SAT).

(1) How do you assess your pain relief after treatment in this study?
□ I feel my pain is much worse (−2)
□ I feel my pain is somewhat worse (−1)
□ I feel my pain is no better and no worse (0)
□ I feel my pain is somewhat better (1)
□ I feel my pain is much better (2)
(2) How do you assess your activity level after treatment in this study?
□ I feel much less active (−2)
□ I feel somewhat less active (−1)
□ I feel no more and no less active (0)
□ I feel somewhat more active (1)
□ I feel much more active (2)
(3) How has your quality of life changed after treatment in this study?
□ I feel my quality of life is much worse (−2)
□ I feel my quality of life is somewhat worse (−1)
□ I feel my quality of life is no better and no worse (0)
□ I feel my quality of life is somewhat better (1)
□ I feel my quality of life is much better (2)
(4) Would you undergo this treatment again?^∗^
□ No, definitely not (−2)
□ No, probably not (−1)
□ Unsure (0)
□ Yes, probably (1)
□ Yes, definitely (2)
(5) How do you compare the treatment you received in this study to previous medication or therapies for your pain?
□ Very much prefer my previous treatments to this treatment (−2)
□ Somewhat prefer my previous treatments (−1)
□ No preference (0)
□ Somewhat prefer this treatment to my previous treatment (1)
□ Very much prefer this treatment to my previous treatments (2)

*In Study C116, SAT Item 4 was administered with 3 response options: “No, absolutely not” (−2), “Unsure” (0), and “Yes, definitely” (2). The item was administered in Study C117 with 5 response levels as shown above.

**Table 2:** Schedule of Study Visits and Measures.

	Screening	Baseline	Visit 1	Visit 2	Termination Visit
	(14+ days)	(Week 0)	(Week 4)	(Week 8)	(Week 12)
Numeric Pain Rating Scale (NPRS)	x	x	x	x	x
Brief Pain Inventory (BPI)	x			x	x
Short-Form McGill Pain Questionnaire (SF-MPQ)	x			x	x
Short Form-36 Heath Survey version 2 (SF-36v2)	x			x	
Patient's Global Impression of Change (PGIC)			x	x	x
Clinician Global Impression of Change (CGIC)^∗^			x	x	x
Self-Assessment of Treatment (SAT)					x

*CGIC was collected in Study C117 only.

**Table 3:** Patient demographic characteristics at screening (pooled dataset; *N* = 698)^1^.

	*N*
Age (years)	
Mean (SD)	70.9 (11.7)
Min, Max	21–94

Age group [years; *n* (%)]
≤50	44 (6.3%)
51–60	72 (10.3%)
61–70	168 (24.1%)
71–80	275 (39.4%)
>80	139 (19.9%)

Sex, *n* (%)
Male	319 (45.7%)
Female	379 (54.3%)

Ethnicity, *n* (%)	
Hispanic or Latino	25 (3.6%)
Not Hispanic or Latino	673 (96.4%)

Race
White	648 (92.8%)
African American	21 (3.0%)
Asian	12 (1.7%)
Other	17 (2.4%)

SD: standard deviation.

^1^Pooled dataset included data from two clinical trials, Studies C116 (*N* = 349) and C117 (*N* = 349).

**Table 4:** Descriptive statistics for PRO measures at screening and baseline (pooled dataset; *N* = 698)^1^.

	*N*	Mean (SD)	Median	Range
NPRS pain now (screening)	698	4.7 (2.2)	5	0.0–10.0

NPRS average pain in last 24 hours^2^ (baseline)	695	5.8 (1.6)	6	1.6–9.9

BPI (screening)
Pain at its worst in the last 24 hours	698	6.8 (2.0)	7	0.0–10.0
Pain at its least in the last 24 hours	698	3.1 (2.1)	3	0.0–10.0
Pain on average in the last 24 hours	698	5.1 (1.7)	5	0.0–10.0
Pain right now	698	4.5 (2.3)	4	0.0–10.0

BPI pain interference scores (screening)
A. General activity	698	3.6 (2.9)	3	0.0–10.0
B. Mood	697	3.7 (2.8)	3	0.0–10.0
C. Walking ability	697	2.5 (2.9)	1	0.0–10.0
D. Normal work	697	3.6 (2.9)	3	0.0–10.0
E. Relation with other people	698	2.5 (2.6)	2	0.0–10.0
F. Sleep	698	4.1 (3.1)	4	0.0–10.0
G. Enjoyment of life	698	4.2 (2.9)	4	0.0–10.0

SF-MPQ (screening)
Present pain intensity	696	2.1 (0.9)	2	0.0–5.0

SF-36v2 (screening)
Physical functioning	698	59.5 (27.9)	60	0.0–100.0
Role-physical	698	55.6 (28.0)	56	0.0–100.0
Bodily pain	698	44.0 (18.9)	41	0.0–100.0
General health	698	67.4 (19.3)	67	5.0–100.0
Role-emotional	698	71.9 (27.3)	75	0.0–100.0
Vitality	698	52.0 (20.7)	56	0.0–100.0
Mental health	698	71.8 (18.7)	75	5.0–100.0
Social functioning	698	71.6 (26.2)	75	0.0–100.0

PGIC (Week 12)	697	0.9 (1.2)	1	−3.0–3.0

CGIC^3^ (Week 12)	349	1.0 (1.2)	1	−2.0–3.0

BPI: Brief Pain Inventory; CGIC: Clinician Global Impression of Change; NPRS: Numeric Pain Rating Scale; PGIC: Patient Global Impression of Change; SD: standard deviation.

^1^Pooled dataset included data from two clinical trials, Studies C116 (*N* = 349) and C117 (*N* = 349).

^2^Average NPRS pain rating for 7 days prior to visit.

^3^Study C117 only.

**Table 5:** Descriptive statistics for SAT items at Week 12 (pooled dataset; *N* = 698)^1^.

SAT item	*N*	Mean (SD)	Median	Floor (*n*, %)	Ceiling (*n*, %)
(1) How do you assess your pain relief after treatment in this study?	698	0.6 (0.9)	0	8 (1.1%)	154 (22.1%)
(2) How do you assess your activity level after treatment in this study?	698	0.4 (0.8)	0	8 (1.1%)	86 (12.3%)
(3) How has your quality of life changed after treatment in this study?	698	0.5 (0.8)	0	6 (0.9%)	114 (16.3%)
(4) Would you undergo this treatment again?	698	1.0 (1.2)	2	41 (5.9%)	356 (51.0%)
(5) How do you compare the treatment you received in this study to previous medication or therapies for your pain?	698	0.5 (1.1)	0	39 (5.6%)	179 (25.6%)

SAT: Self-Assessment of Treatment; SD: standard deviation.

^1^Pooled dataset included data from two clinical trials, Studies C116 (*N* = 349) and C117 (*N* = 349).

**Table 6:** Standardized factor loadings for one- and two-tactor exploratory and confirmatory SAT models (pooled dataset; *N* = 698)^1^.

SAT item	Factor loadings				
	1-factor EFA/CFA	2-factor EFA		2-factor CFA	
		Domain 1	Domain 2	Domain 1	Domain 2
(1) How do you assess your pain relief after treatment in this study?	0.83	0.65	0.24	0.82	
(2) How do you assess your activity level after treatment in this study?	0.85	0.89	−0.05	0.85	
(3) How has your quality of life changed after treatment in this study?	0.92	0.93	0.01	0.93	
(4) Would you undergo this treatment again?	0.47	−0.01	0.66		0.59
(5) How do you compare the treatment you received in this study to previous medication or therapies for your pain?	0.65	0.21	0.62		0.84

EFA: exploratory factor analysis; CFA: confirmatory factor analysis; SAT: Self-Assessment of Treatment.

^1^Exploratory and confirmatory factor analyses were conducted to evaluate the factor (scale) structure of the SAT and fit of the 5 items within the hypothesized scale. Pooled dataset included data from two clinical trials, Studies C116 (*N* = 349) and C117 (*N* = 349).

**Table 7:** Construct validity: correlations between SAT and PRO measures (pooled dataset; *N* = 698)^1^.

				Spearman correlations^2^			
	SAT 1 (Pain relief)	SAT 2 (Activity level)	SAT 3 (Quality of life)	SAT 4 (Undergo treatment again)	SAT 5 (Compared to previous treatment)	3-Item SAT treatment effect subscale	2-Item SAT treatment satisfaction subscale
PGIC	0.90	0.68	0.77	0.44	0.62	0.89	0.61

CGIC^3^	0.85	0.63	0.73	0.48	0.64	0.83	0.64

NPRS change scores
Pain now	−0.60	−0.47	−0.51	−0.30	−0.37	−0.59	−0.38
Average 24 hour pain	−0.69	−0.53	−0.60	−0.35	−0.45	−0.68	−0.46

BPI change scores
Pain in last 24 hours—at worst	−0.64	−0.50	−0.53	−0.28	−0.40	−0.63	−0.39
Pain in last 24 hours—at least	−0.52	−0.38	−0.44	−0.27	−0.32	−0.50	−0.34

SF-MPQ present pain intensity change scores	−0.45	−0.41	−0.43	−0.20	−0.29	−0.47	−0.29

SF-36v2 change scores
Physical functioning	0.20	0.16	0.25	0.14^4^	0.15^4^	0.23	0.16
Bodily pain	0.43	0.40	0.40	0.27	0.29	0.46	0.33
Vitality	0.25	0.23	0.21	0.09^5^	0.14^4^	0.25	0.14^4^

BPI: Brief Pain Inventory; CGIC: Clinician Global Impression of Change; NPRS: Numeric Pain Rating Scale; PGIC: Patient Global Impression of Change; SAT: Self-Assessment of Treatment.

^1^Construct validity of SAT item and subscales were evaluated by examining relationships between SAT items and subscales with component scores of conceptually-related outcome measures using Spearman correlation coefficients. Pooled dataset included data from two clinical trials, Studies C116 (*N* = 349) and C117 (*N* = 349).

^2^Bivariate correlations were significant at *P* < 0.0001, except where noted otherwise.

^3^Study C117 only.

^4^ *P* < 0.001.

^5^ *P* < 0.05.

**Table 8:** Known-groups validity: ANOVA models of SAT scores by global assessment levels at week 12 (pooled dataset; *N* = 698)^1^.

					SAT at Week 12			
	*n*	(1) Pain relief	(2) Activity level	(3) Quality of life	(4) Undergo treatment again	(5) Compared to previous treatment	3-Item SAT treatment effect subscale	2-Item SAT treatment satisfaction subscale
PGIC, LS mean (SE)								
Very much improved	103	1.9 (0.0)	1.4 (0.1)	1.7 (0.1)	1.7 (0.1)	1.7 (0.1)	5.0 (0.1)	3.5 (0.2)
Much improved	115	1.4 (0.0)	0.8 (0.1)	1.0 (0.0)	1.6 (0.1)	1.1 (0.1)	3.3 (0.1)	2.7 (0.1)
Slightly improved	159	0.8 (0.0)	0.3 (0.0)	0.5 (0.0)	1.1 (0.1)	0.5 (0.1)	1.6 (0.1)	1.6 (0.1)
No change	275	0.0 (0.0)	0.0 (0.0)	0.0 (0.0)	0.6 (0.1)	0.0 (0.1)	−0.1 (0.1)	0.6 (0.1)
Slightly worse	31	−0.7 (0.1)	−0.3 (0.1)	−0.1 (0.1)	0.0 (0.2)	−0.2 (0.2)	−1.1 (0.2)	−0.2 (0.3)
Much worse^2^	14	−1.2 (0.1)	−0.9 (0.2)	−0.9 (0.1)	−0.9 (0.3)	−1.1 (0.2)	−3.0 (0.3)	−2.0 (0.4)
*P*-value	<0.0001	<0.0001	<0.0001	<0.0001	<0.0001	<0.0001	<0.0001

CGIC^3^, LS mean (SE)								
Very much improved	63	1.9 (0.1)	1.4 (0.1)	1.7 (0.1)	1.7 (0.1)	1.7 (0.1)	5.0 (0.2)	3.5 (0.2)
Much improved	56	1.4 (0.1)	0.7 (0.1)	1.0 (0.1)	1.3 (0.1)	1.1 (0.1)	3.0 (0.2)	2.4 (0.2)
Slightly improved	78	0.7 (0.1)	0.3 (0.1)	0.5 (0.1)	0.9 (0.1)	0.4 (0.1)	1.5 (0.2)	1.3 (0.2)
No change	140	−0.1 (0.0)	−0.1 (0.1)	−0.1 (0.0)	0.3 (0.1)	−0.2 (0.1)	−0.3 (0.1)	0.1 (0.1)
Slightly worse	10	−0.1 (0.2)	0.1 (0.2)	0.2 (0.2)	−0.2 (0.3)	−0.1 (0.3)	0.2 (0.4)	−0.3 (0.5)
Much worse^2^	2	−1.5 (0.4)	−0.5 (0.5)	−0.5 (0.4)	−1.5 (0.7)	−0.5 (0.7)	−2.5 (1.0)	−2.0 (1.1)
*P*-value		<0.0001	<0.0001	<0.0001	<0.0001	<0.0001	<0.0001	<0.0001

ANOVA: analysis of variance; CGIC: Clinician Global Impression of Change; LS: least-squares; PGIC: Patient Global Impression of Change; SAT: Self-Assessment of Treatment; SE: standard error.

^1^Known-groups validity was assessed using ANOVA to evaluate whether SAT items and subscale scores discriminated between patient groups based on different amounts of patient- and clinician-reported change at Week 12. Pooled dataset included data from two clinical trials, Studies C116 (*N* = 349) and C117 (*N* = 349).

^2^PGIC and CGIC responses of “Very much worse” and “Much worse” were combined due to small sample sizes.

^3^Study C117 only.

**Table 9:** Known-groups validity: *t*-tests of SAT scores by NPRS pain level at baseline (pooled dataset; *N* = 698)^1^.

SAT	Baseline NPRS pain level		
	High pain (*n* = 172)	Low pain (*n* = 526)	*P*-value
	Mean (SD)	Mean (SD)	
(1) Pain relief	0.4 (1.0)	0.7 (0.9)	0.001
(2) Activity level	0.2 (0.8)	0.4 (0.8)	0.013
(3) Quality of life	0.4 (0.8)	0.5 (0.8)	0.015
(4) Undergo treatment again	1.0 (1.2)	1.0 (1.2)	0.841
(5) Compared to previous treatment	0.4 (1.1)	0.5 (1.1)	0.080
SAT treatment effect subscale (Items 1, 2, and 3)	1.0 (2.3)	1.6 (2.3)	0.002
SAT treatment satisfaction subscale (Items 4 and 5)	1.3 (1.9)	1.5 (2.0)	0.275

NPRS: Numeric Pain Rating Scale; SAT: Self-Assessment of Treatment; SD: standard deviation.

^1^Known-groups validity was assessed using *t*-tests to evaluate whether SAT items and subscale scores discriminated between patient groups by baseline pain level (high, low pain). Pooled dataset included data from two clinical trials, Studies C116 (*N* = 349) and C117 (*N* = 349).

**Table 10:** Concurrent validity: ANOVA models of NPRS change scores by SAT scores at Week 12 (pooled dataset; *N* = 698)^1^.

SAT		NPRS change scores at Week 12					
		Pain now			Average pain in last 24 hours		
		*N*	LS mean (SE)	*P*-value	*N*	LS mean (SE)	*P*-value
(1) How do you assess your pain level after treatment in this study?	Better	341	−2.5 (0.1)	<0.0001	339	−2.9 (0.1)	<0.0001
	No change	310	−0.3 (0.1)	303	−0.5 (0.1)
	Worse	47	0.8 (0.3)	46	(0.2)
(2) How do you assess your activity level after treatment in this study?	Better	210	−2.9 (0.1)	<0.0001	209	−3.2 (0.1)	<0.0001
	No change	453	−0.7 (0.1)	445	−1.0 (0.1)
	Worse	35	0.4 (0.4)	34	−0.1 (0.3)
(3) How has your quality of life changed after treatment in this study?	Better	266	−2.6 (0.1)	<0.0001	265	−3.1 (0.1)	<0.0001
	No change	410	−0.6 (0.1)	402	−0.8 (0.1)
	Worse	22	1.2 (0.4)	21	(0.4)
(4) Would you undergo this treatment again?	Better	451	−1.8 (0.1)	<0.0001	445	−2.1 (0.1)	<0.0001
	No change	174	−0.4 (0.2)	172	−0.8 (0.1)
	Worse	73	−0.3 (0.3)	71	−0.5 (0.2)
(5) How do you compare the treatment you received in this study to previous medication or therapies for your pain?	Better	296	−2.2 (0.1)	<0.0001	294	−2.6 (0.1)	<0.0001
	No change	312	−0.7 (0.1)	306	−0.9 (0.1)
	Worse	90	−0.4 (0.2)	88	−0.8 (0.2)
SAT treatment effect subscale (Items 1, 2, and 3)	Better	359	−2.4 (0.1)	<0.0001	357	−2.8 (0.1)	<0.0001
	No change	280	−0.3 (0.1)	273	−0.5 (0.1)
	Worse	59	0.9 (0.3)	58	0.2 (0.2)
SAT treatment satisfaction subscale (Items 4 and 5)	Better	448	−1.8 (0.1)	<0.0001	443	−2.1 (0.1)	<0.0001
	No change	141	−0.5 (0.2)	138	−0.9 (0.2)
	Worse	109	−0.2 (0.2)	107	−0.5 (0.2)

ANOVA: analysis of variance; LS: least-squares; NPRS: Numeric Pain Rating Scale; SAT: Self-Assessment of Treatment; SE: standard error.

^1^Concurrent validity was evaluated using ANOVA to compare NPRS changes in pain by SAT response levels (patients improved; no change; worsened). Pooled dataset included data from two clinical trials, Studies C116 (*N* = 349) and C117 (*N* = 349).

**Table 11:** Concurrent validity: ANOVA models of BPI change scores by SAT scores at Week 12 (pooled dataset; *N* = 698)^1^.

SAT		BPI change scores at Week 12					
		Pain at its worst in last 24 hours			Pain at its least in the last 24 hours		
		*N*	LS mean (SE)	*P*-value	*N*	LS mean (SE)	*P*-value
(1) How do you assess your pain level after treatment in this study?	Better	340	−3.6 (0.1)	<0.0001	340	−1.3 (0.1)	<0.0001
	No change	309	−0.8 (0.1)	309	0.7 (0.1)
	Worse	47	0.5 (0.3)	47	1.3 (0.3)
(2) How do you assess your activity level after treatment in this study?	Better	210	−4.1 (0.2)	<0.0001	210	−1.5 (0.1)	<0.0001
	No change	451	−1.3 (0.1)	451	0.3 (0.1)
	Worse	35	0.2 (0.4)	35	(0.4)
(3) How has your quality of life changed after treatment in this study?	Better	266	−3.8 (0.1)	<0.0001	266	−1.4 (0.1)	<0.0001
	No change	408	−1.1 (0.1)	408	0.5 (0.1)
	Worse	22	0.3 (0.5)	22	(0.4)
(4) Would you undergo this treatment again?	Better	450	−2.6 (0.1)	<0.0001	450	−0.6 (0.1)	<0.0001
	No change	173	−1.1 (0.2)	173	0.5 (0.2)
	Worse	73	−0.9 (0.3)	73	(0.3)
(5) How do you compare the treatment you received in this study to previous medication or therapies for your pain?	Better	295	−3.3 (0.1)	<0.0001	295	−1.1 (0.1)	<0.0001
	No change	312	−1.3 (0.1)	312	0.4 (0.1)
	Worse	89	−1.0 (0.3)	89	0.3 (0.2)
SAT treatment effect subscale (Items 1, 2, and 3)	Better	358	−3.5 (0.1)	<0.0001	358	−1.2 (0.1)	<0.0001
	No change	279	−0.8 (0.1)	279	0.7 (0.1)
	Worse	59	0.4 (0.3)	59	1.2 (0.3)
SAT treatment satisfaction subscale (Items 4 and 5)	Better	447	−2.7 (0.1)	<0.0001	447	−0.6 (0.1)	<0.0001
	No change	141	−1.0 (0.2)	141	0.6 (0.2)
	Worse	108	−0.9 (0.3)	108	0.5 (0.2)

ANOVA: analysis of variance; BPI: Brief Pain Inventory; LS: least-squares; SAT: Self-Assessment of Treatment; SE: standard error.

^1^Concurrent validity was evaluated using ANOVA to compare BPI changes in pain by SAT response levels (patients improved; no change; worsened). Pooled dataset included data from two clinical trials, Studies C116 (*N* = 349) and C117 (*N* = 349).
